# Public perceptions of myocardial infarction: Do illness perceptions predict preferences for health check results

**DOI:** 10.1016/j.pmedr.2021.101683

**Published:** 2022-01-24

**Authors:** Åsa Grauman, Jennifer Viberg Johansson, Marie Falahee, Jorien Veldwijk

**Affiliations:** a Centre for Research Ethics & Bioethics, Uppsala University, Uppsala, Sweden; b The Institute of Future Studies, Stockholm, Sweden; c Rheumatology Research Group, Institute of Inflammation and Ageing, University of Birmingham, Birmingham, UK; d Erasmus School of Health Policy & Management, Erasmus University, Rotterdam, Netherlands; e Erasmus Choice Modelling Centre, Erasmus University, Rotterdam, Netherlands

**Keywords:** Myocardial infarction, Illness perception, Causal beliefs, Public, Preferences, Health check, Discrete choice experiment

## Abstract

Illness perceptions are associated with attitudes towards preventive behaviors and are therefore crucial to consider in the context of prevention of cardiovascular diseases. We investigated illness perceptions of the public about myocardial infarction, and whether they predict public preferences for health check test results.

A randomly selected sample (N = 423) of the Swedish public aged 40–70 completed an online-survey. It included the brief illness perception questionnaire, items assessing sociodemographic, lifestyle and health factors and a discrete choice experiment incorporating six attributes of health checks (written results, notification method, consultation time, waiting time, lifestyle recommendation and cost). Associations between illness perceptions and sociodemographic- and cardiovascular risk factors were analyzed using multivariate linear regression. Preference data were analyzed with a mixed multinomial logit model.

Presence of smoking, hypertension, obesity and lack of physical activity were associated with weaker causal beliefs for the relevant risk factor, while presence of a high stress level was associated with stronger causal beliefs for stress. Low control predicted unwillingness to receive lifestyle recommendations. Attributing family history as the most important personal cause of MI predicted unwillingness to participate in health checks.

Illness perceptions differed due to presence of risk factors, age, sex and health literacy. Furthermore, illness perceptions influenced preferences for health check test results and willingness to participate in health checks. Illness perceptions should therefore be addressed when designing health communication and preventive interventions such as health checks, and methods for promoting accurate illness perceptions should be developed.

## 1 Introduction

Cardiovascular diseases (CVD) have multifactorial causes, many of which are modifiable by lifestyle changes and preventive treatment, and are managed by the individual in her/his daily life (Yusuf et al., 2004). The development of CVD may be asymptomatic, and the first sign can be a serious event such as a myocardial infarction (MI). Confusion around CVDs as chronic illnesses while MI as an acute event, together with the multifactorial aetiology of CVD, make the disease a complex concept, that may be difficult for people to understand (Angus et al., 2005).

The Common-Sense Model of Self-Regulation (CSM) is a theoretical framework developed by Leventhal et al, for understanding and explaining how people make sense and respond to health threats. It is useful for predicting self-management. It can also be useful for creating health- and risk communication (Leventhal et al., 2016). It is therefore relevant to consider in prevention and management of chronic diseases, such as CVD (Leventhal et al., 1992). CSM describes how a person forms cognitive and emotional representations of an illness, and develops coping behaviors that are based on the perceived and experienced efficiency of these behaviors. Illness representations are influenced by personal experiences of illness and treatments, but also through observing others and communication from close-ones, health care services and mass media (Leventhal et al., 2016). Five dimensions originally described the cognitive representation of an illness: identity (symptoms), consequences (effect and outcomes), cause (what causes the illness), timeline (how long it will last) and control (beliefs about individuals own control over the illness). Emotional representations involve negative emotions such as fear, anger and distress (Leventhal et al., 1998). Later, coherence (understanding), personal control and treatment control were added to the model (Broadbent et al., 2015).

Illness perceptions about CVD have been found to be associated with BMI, blood pressure (Petriček et al., 2009), and HRQL (Flora et al., 2015, Alsén et al., 2010). Exercise adherence and treatment adherence was predicted by a strong perception of control, while adherence to a low-fat diet was predicted by illness coherence (Mosleh and Almalik, 2016). Attendance in cardiac rehabilitation groups was associated with several illness perception dimensions. Patients who perceived their condition as controllable, as symptomatic, and with severe consequences, and who felt that they understand their condition were more likely to attend rehabilitation (Flora et al., 2015, French et al., 2006).

Regarding beliefs about causality, patients not attending cardiac rehabilitation were more likely to attribute their illness to non-modifiable factors (Blair et al., 2014). The majority of patients in a Jordanian sample thought that the cause of their heart problem was related to coronary heart disease risk factors such as obesity and high-fat meals (Mosleh and Almalik, 2016). A systematic review concluded that men are more likely to attribute lifestyle to their CVD, while women more often report psychological factors and hereditary conditions as causes of their CVD (Al-Smadi et al., 2016). Causal beliefs have differed between patients and the public, where patients were more likely to attribute their illness to stress and bad luck, while non-patients perceived overweight and hypertension as causes that are more important (French et al., 2001).

Research about CSM has mainly been applied in patient populations. However, in recent years there have been examples in the context of healthy members of the public (Figueiras and Alves, 2007). For instance, illness perceptions were associated breast cancer screening attendance in Malta (Marmarà et al., 2017), and COVID-19 vaccination willingness among young Dutch citizens (Vollmann and Salewski, 2021). To our awareness CSM have not been used to explain willingness to participate in health checks, which are a strategy to prevent CVDs within the public. A previous study of public preferences for health check test results, found differences in preferences, and in the overall willingness to participate in the health checks (Grauman et al., 2021). Explaining such differences is important in order to improve the satisfaction and uptake-rates of public health checks. Psychological and cognitive factors such as illness perceptions may be important modifiable determinants of choice (Russo et al., 2019). Furthermore, knowledge about the publics’ illness beliefs of CVD, is important to build future risk and health information upon. The objective of this study was to investigate how the Swedish public perceives MI, and whether illness perceptions are associated with age, sex, education, health literacy, presence of CVD risk factors, and preferences for risk assessment via a health check.

## 2 Material and methods

### 2.1 Study population and data collection

This was a cross-sectional study among a random sample of Swedish citizens, aged 40–70 years (n = 1650), drawn from the national registration register. An invitation letter included information about the study together with a link to an online-survey (Sawtooth Lighthouse Studio 9.7). The respondents were informed that a paper survey could be sent to their home if they preferred. Two reminders were sent after three and six weeks. Data was collected January-March 2020. A description of the development of the discrete choice experiment (DCE), and data collection for this study has been described previously (Grauman et al., 2021). Ethical approval was granted by the Swedish Ethical Review Authority (dnr:2019:03843). Consent to participate was obtained from all respondents. This work has been carried out in accordance with Declaration of Helsinki and prioritized participants privacy and safety.

### 2.2 Variables and instruments

The survey included questions about sociodemographic factors; sex, age, educational level, and medical or care- related training. It also included questions about health status, medical history, and lifestyle (smoking, stress, physical activity and Body Mass Index (BMI kg/m^2^). Risk perception was assessed on a 5-point Likert scale using the question “compared to other people of the same age and sex as you, how do you perceive your risk of having a heart attack in the next ten years?” Scores were collapsed into three categories: lower than others (1–2 points), same as others (3 points) and higher than others (4–5 points). Health literacy was measured using the validated Swedish Functional Health Literacy Scale (Wångdahl and Mårtensson, 2015).

The illness perception questionnaire (IPQ) is a validated instrument to assess illness perceptions for various diseases and conditions (Broadbent et al., 2015, Broadbent et al., 2006). Illness perceptions of MI were measured using the Brief Illness Perception Questionnaire (B-IPQ) (Broadbent et al., 2006), which has been used for patient groups (including following MI), health care workers (Grankvist and Brink, 2009) and the public (Figueiras and Alves, 2007). The Brief IPQ uses a single-item scale approach to assess cognitive and emotional perceptions on a scale from 0 to10 (Broadbent et al., 2006). In this study items on consequences, timeliness, personal control, treatment control, coherence, and concern were included and adapted for the healthy population. Instead of “your illness” we used “a heart attack,” instead of “control of illness” we used “control over risk of experiencing the illness.” Causal attributions were measured, by asking the respondent to rate the extent to which 14 different risk factors cause MI, on a Likert scale ranging from 1 to 5 where 1 represents not at all and 5 represent to a great extent. Causal attributions were also assessed by asking the respondents, which are the three most important causes of MI for them. Respondents could choose risk factors from a pre-defined list or enter an open-ended response.

### 2.3 Discrete choice experiment

The survey also included a DCE, with the aim to investigate preferences for health test results (Lancsar and Louviere, 2008). The attributes in the DCE were chosen using a step-wise manner involving a literature of previous research, discussion with three experts (physicians and researchers) to ensure the attributes were consistent with current practice and the results of three focus groups with members of the public (Hiligsmann, 2013). Six attributes with appropriate levels were selected. Results format, waiting time, consultation time and lifestyle recommendations were ranked most highly while cost and notification method were chosen due to their policy relevancy (Table 1). NGene 1.0 software was used to generate a Bayesian D-efficient design using best guess priors which minimizes the sample size and the number of choice tasks every respondent is asked to complete (Hensher et al., 2015, Rose and Bliemer, 2009). The design included an interaction effect between lifestyle recommendations and consultation time.

**Table 1:** Attributes and levels included in the DCE.

Attributes	Levels
Written results: How your test results are presented to you in a written format.	Numerical test results with reference values of what is considered normal for the population
	Numerical test results and Everyday words. Besides numerical values, your test result is also presented in everyday words
	Numerical test results, Everyday words and Overall assessment. Besides numerical values and everyday words, your test result include an overall assessment where all test results are included, as well as lifestyle factors and individual factors such as age and sex.
Notification method: Your test results are documented in your medical health record. You can access your test results by logging in to your electronic health record online.	Only electronic health record. You will receive your written test result only by personally logging in to your electronic health record.
	Electronic health record + letter. Besides having access to your written test results through your electronic, you will also receive a letter to your home address or e-mail.
Waiting time: How long you will have to wait for your written test results.	2 days
	1 week
	2 weeks
	3 weeks
Lifestyle recommendations: There are actions you can take yourself to influence your cardiovascular risk, thing related to your lifestyle.	No, lifestyle recommendations are not included
	Yes, lifestyle recommendations are included
Consultation time: Time with a medically trained person with high competence within the area, to get the opportunity to discuss and ask questions regarding your test results.	No consultation time. You will only receive written results.
	15 min. Face-to-face or over the phone
	30 min. Face-to-face or over the phone
Cost*: What you pay out of your own pocket	Free of charge
	€ 15 (150 SEK)
	€ 30 (300 SEK)
	€ 60 (600 SEK)
	€ 90 (900 SEK)
	€ 120 (1200 SEK)

*In the Swedish version, only SEK was presented.

Sixty unique choice tasks were generated. To limit the burden on respondents, the choice tasks were divided into four blocks of 15 choice tasks. Respondents were randomized to one of the blocks. Each choice task consisted of two alternative health check options. After that, respondents were asked if they would actually participate in the selected health check in a real life setting or whether they preferred to opt-out.

Before respondents were asked to complete the choice tasks, they received detailed information on the meaning of the attributes and levels as well as an example of how to complete a choice task. In addition, the context of the health check in the DCE was explained before the choice tasks; *“Imagine being invited to a health check by the public health care authorities. The health check would take place at the local Primary Health Care Center. The testing would include analysis of glucose, blood lipids, blood pressure, as well as measurement of waist circumference, height and weight. You would also be asked to fill out a survey regarding your lifestyle habits. In the following DCE, imagine that no serious clinical findings were detected, but in any such case, you would be referred to health care immediately.”*

The survey was pilot tested with respondents from the target population (n = 32). In addition, three think-aloud interviews were conducted. The pilot resulted in minor changes to the wording of the survey. Based on the pilot-test data, priors were updated to improve the efficiency of the experimental design used for the final DCE. An example of a choice task is presented in Supplementary file, Fig. 1.

**Fig. 1:**
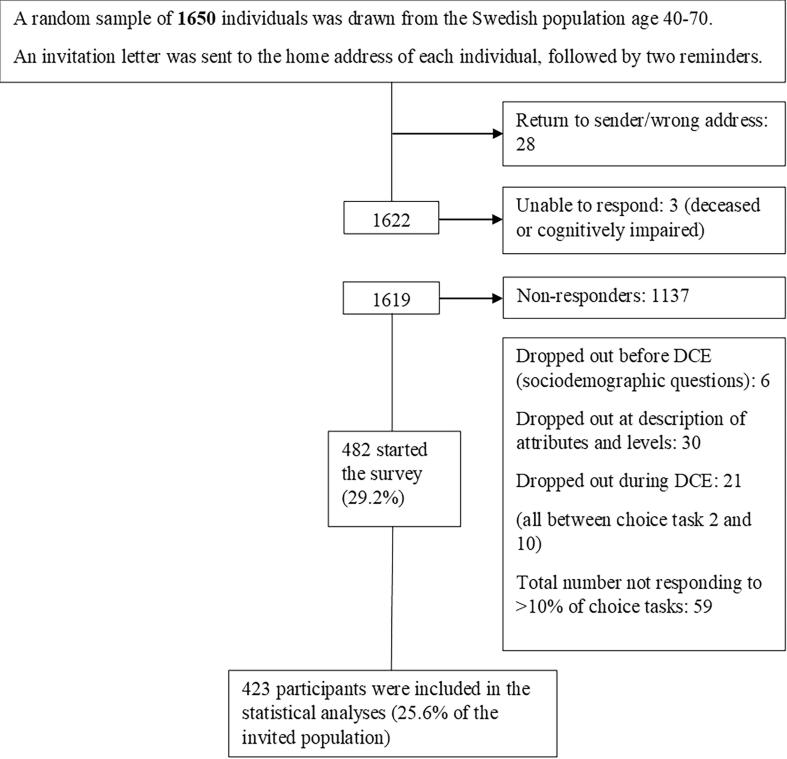
Flowchart of Study population.

### 2.4 Statistical analysis

Descriptive statistics are presented with mean and standard deviation for continuous variables and as frequencies for categorical variables. Associations between illness representations and sociodemographic factors, health literacy, medical training and CVD risk factors, were assessed by t-tests and Spearman’s correlations. Statistically significant findings (p < 0.05) were tested in separate linear regression models for each dimension and adjusted for age, sex and education. Differences in beliefs about causal attribution of CVD risk factors due to sociodemographic variables and the presence of the specific CVD factor (when data was available) were assessed by *t*-test and Spearman’s correlation. BMI was used as a proxy for dietary habits. Statistically significant findings were further tested in separate multivariate linear regression models for each CVD risk factor and adjusted for age, sex and education. Results with a probability < 0.05 were interpreted as statistically significant.

Preference data were analyzed using a mixed multinomial logit model, which takes into account possible preference heterogeneity and adjusts for the multilevel structure (one respondent makes more than one choice) of the data. The opt-out estimates the a priori preference of respondents declining a health check. (i.e., choosing to opt-out) over accepting the health check. Effects coding was used for the attribute levels, which allows estimation of all attribute level effects. Interactions were introduced into the model by combining illness perceptions variables (dichotomized using a median split), ratings of causal beliefs and personal causal beliefs with the attribute levels and constant. The interactions were first added separately, after which all significant effects were combined into one model. The interactions included in the final model were decided based on statistical significance level (*p* < 0.05) and overall model fit (AIC).

## 3 Results

In total, 423 respondents completed the survey, and were included in the statistical analyses (Fig. 1), flowchart of study population), response rate = 25.6%. Characteristics of the respondents are presented in Table 2. Overweight was the most common CVD risk factor (70%), followed by hypertension (30%), while only 5% of the respondents smoked.

**Table 2:** Characteristics of study population. N = 423.

Variable (n responses)		
	n (%)	Mean (SD)
Age (423)		57.3 (8.7)
Sex (422)		
*Female*	209 (49.5)	
*Male*	213 (50.5)	
*Other*	-	
Education (419)		
*Primary school*	31 (7.4)	
*High school*	187 (44.6)	
*University*	201 (47.5)	
Health literacy (421)		
*High*	132 (31.4)	
*Moderate*	176 (41.8)	
*Low*	113 (26.8)	
Medical or care related training (421)		
*yes*	84 (19.9)	
Family history of MI (411)		
*Yes*	103 (25.1)	
BMI (405)		26.3 (4.3)
≥25 (Overweight)	283 (69.9)	
≥30 (Obesity)	97 (24.0)	
Smoking (417)		
Yes	21 (5.0)	
Experienced stressful period (416)		
Low level:	321 (75.9)	
*Never*	*21 (5.0)*	
*Some periods*	*117 (28.1)*	
*Some periods the last 5 years*	*183 (44.0)*	
High level:	95 (22.5)	
*Constant stress*	*44 (10.6)*	
*Constant stress the last 5 years*	*51 (12.3)*	
Physical activity (418)		
*Never*	77 (18.2)	
*Time to time*	72 (17.0)	
*1*–*2/week*	98 (23.4)	
*2*–*3/week*	92 (22.0)	
*>3 week*	79 (18.9)	
Hypertension (treated or diagnosed) (423)	119 (28.1)	
Cholesterol (treated or diagnosed) (423)	62 (14.7)	
Diabetes(treated or diagnosed) (423)	30 (7.1)	
CVD (treated or diagnosed) (423)	30 (7.1)	
Risk perception (399)		
*Lower than others*	156 (39.1)	
*Same as others*	169 (42.4)	
*Higher than others*	74 (18.6)	

### 3.1 Illness representations

The respondents’ illness representations of MI are presented in Table 3; consequences was rated highest while concern was rated lowest. Differences in illness representations due to sociodemographic factors, health literacy, medical/caring training and CVD risk factors were tested in univariate analyses (Supplement Table 1). Statistical significant variables were further tested in multivariate linear regression models and adjusted for age, sex and education.

**Table 3:** Mean and standard deviation (SD) of illness representations. Standardized beta coefficient for association of causal beliefs with CVD risk factors, sex, age, education and health literacy (only variables that were statistically significant in univariate analyses were included). Each dimension was tested in a separate model.

Illness representations (n) CVD risk factors	Mean (SD)	β (CI) crude	β (CI) adjusted for age, sex, education
Consequence (384)	8.2 (1.7)		
Smoker		−0.12 *(−1.85; −0.15)	−0.12* (−1.89; −0.19)
Family history of MI		−0.11* (−0.85; −0.03)	−0.11* (−0.84; −0.02)

Timeliness (360)	6.8 (2.0)		
High stress		0.13* (0.12; 1.11)	0.15** (0.21; 1.23)
Diagnosed or treated with diabetes		0.13* (0.17; 1.80)	0.12* (0.09; 1.75)

Personal control (395)	5.8 (2.3)		
Risk perception (higher)		−0.11* (−1.25; −0.05)	−0.11* (−1.22; −0.02)
High stress		−0.11* (−1.12; −0.03)	−0.12* (−1.20; −0.06)
Smoker		−0.15** (−2.67; −0.50)	−0.14** (−2.59; −2.59)

Treatment control (358)	6.0 (2.3)		
Diagnosed or treated with hypertension		0.11 (0.00; 1.05)	0.10 (−0.05; 1.05)
Diagnosed or treated with CVD		0.14* (0.28; 2.10)	0.13* (0.25; 2.09)

Concern, Worry (414)	2.8 (2.5)		
High stress		0.12* (0.16; 1.32)	0.14** (0.25; 1.44)
BMI obese		0.03 (−0.50; 0.83)	0.01 (−0.61; 0.73)
Family history of MI		0.16** (0.39; 1.52)	0.15** (0.29; 1.44)
Diagnosed or treated with CVD		0.11* (0.13; 2.01)	0.10 (0.00; 1.91)
Diagnosed or treated with hypertension		0.18*** (0.48; 1.56)	0.17** (0.38; 1.50)
Risk perception (higher)		0.27*** (1.11; 2.34)	0.26*** (1.04; 2.27)
Health literacy (high)		−0.15** (−1.33; −0.27)	−0.14** (−1.28; −0.22)
Age^a^		0.08 (−0.00; 0.05)	0.07 (−0.01; 0.05)

Coherence (396)	5.4 (2.5)		
Family history		0.14** (0.23; 1.41)	0.13* (0.15; 1.34)
Diagnosed or treated with CVD		0.13* (0.26; 2.25)	0.12* (0.16; 2.17)
Medical or caring training		0.29*** (2.11; 3.26)	0.32*** (2.18; 3.44)
Health literacy (high)		0.10* (0.03; 1.11)	0.10 (−0.02; 1.06)
Sex^b^ (female)		0.11* (0.04; 1.05)	0.10 (−0.02; 0.99)
Age^a^		0.10 (0.00; 0.06)	0.11 (0.00; 0.06)

a only adjusted for sex and education ^b^ only adjusted for age and education *p < 0.05 **p < 0.01 ***p < 0.001.

Respondents with family history of MI and smokers perceived the consequences of MI to be less severe compared to others. Respondents with diabetes and high stress believed that the illness would last longer. Respondents that perceived themselves to be at higher risk of MI than their peers, smokers and respondents with high stress level experienced less personal control. Respondents with CVD had stronger beliefs about the effectiveness of drug treatment to prevent MI. Respondents with CVD, hypertension, higher stress, family history of MI, low/moderate health literacy, and respondents who perceived their risk as higher, reported higher levels of concern. The strongest correlation with concern was with risk perception. Respondents with medical/caring training, CVD and a family history of MI reported higher understanding of MI (Table 3).

### 3.2 Causal attribution

Smoking, hypertension and obesity were most often reported to be the causes of MI. Virus/bacteria and diabetes were the causes with the highest numbers of “don’t know” responses, while a small number of respondents answered “don’t know” about obesity, diet and physical activity (Table 4). Respondents without medical or care related training and respondents with low educational level, more often responded “don’t know” (*p* < 0.05).

**Table 4:** Causal attribution. How much do you think the following factor contributes to causing MI? 1 (not at all) − 5 (extremely much). N = 421.

CVD risk factors	Mean (SD)	Do not know n (%)
Smoking	4.4 (0.8)	10 (2.4)
Hypertension	4.2 (0.8)	10 (2.4)
Overweight/obesity	4.2 (0.8)	3 (0.7)
High cholesterol	4.1 (0.8)	13 (3.1)
Stress	4.1 (0.8)	12 (2.9)
Unhealthy diet	4.0 (0.8)	4 (1.0)
Lack of PA	3.9 (0.9)	5 (1.2)
Heritage, it runs in the family	3.8 (0.9)	12 (2.9)
Diabetes	3.6 (0.97)	98 (23.3)
High alcohol intake	3.6 (0.9)	38 (9.0)
Ageing	3.3 (0.9)	24 (5.7)
Worry, sadness, loneliness	3.1 (1.1)	43 (10.2)
Virus/bacteria	2.4 (1.2)	129 (30.6)
Bad luck/chance	2.2 (1.0)	60 (14.3)

Differences in causal attributions due to age, sex, education, health literacy and presence of specific risk factors (for variables were information was available) were tested in univariate analyses (Supplement Table 2). Statistically significant associations were further tested for the different causal beliefs in linear regression adjusted for sex, age and education (Table 5). Older respondents had stronger causal beliefs about smoking and worry, sadness, loneliness compared with younger respondents. Female respondents had stronger causal beliefs about stress and lack of physical activity than men. No differences in causal beliefs were found for educational level. Respondents with high health literacy had stronger causal beliefs about smoking, hypertension, unhealthy diet and lack of physical activity than respondents with moderate or low health literacy.

**Table 5:** Standardized beta coefficient with 95% confidence interval (CI), for association of causal beliefs with presence of specific risk factor, sex, age, education and health literacy (only variables that were statistically significant in univariate analyses were included). Each risk factor were tested in a separate model.

Causal attributions	β (CI)crude	β (CI) adjusted for age, sex, education
Smoking		
Presence of smoking	−0.18*** (−0.99; −0.30)	−0.19*** (−1.02; −0.34)
Health literacy (high)	0.11* (0.02; 0.35)	0.11* (0.02; 0.35)
Age^a^	0.15** (0.01; 0.02)	0.16** (0.01; 0.02)

Hypertension		
Presence of hypertension	−0.16** (−0.46; −0.11)	−0.18*** (−0.51; −0.15)
Health literacy (high)	0.13* (0.05; 0.39)	0.12* (0.04; 0.38)

Overweight/obesity		
Presence of obesity	−0.16** (−0.54; −0.13)	−0.16** (−0.54; −0.12)

Unhealthy diet		
Presence of obesity	−0.11* (−0.46; −0.03)	−0.11 (−0.03; −0.00)
Health literacy	0.12* (0.03; 0.38)	0.11* (0.02; 0.36)
Stress		
Presence of high stress	0.10* (0.01; 0.39)	0.12* (0.03; 0.43)
Sex^b^ (female)	0.12* (0.04; 0.36)	0.12* (0.03; 0.36)

Lack of physical activity		
Presence of lack of PA	−0.13** (−0.41; −0.07)	−0.13*** (−0.42; −0.07)
Health literacy (high)	0.12* (0.03; 0.39)	0.12* (0.03; 0.39)

Worry, loneliness, sadness		
Age^a^	0.22*** (0.02; 0.04)	0.23*** (0.02; 0.04)

a only adjusted for sex and education ^b^ only adjusted for age and education *p < 0.05 **p < 0.01 ***p < 0.001.

Respondents, who themselves smoked, or never exercised, or were treated or diagnosed with hypertension or were obese, reported weaker causal beliefs of the specific risk factor. Likewise, respondents with obesity reported weaker causal beliefs for unhealthy diet. For stress, the opposite was found; respondents experiencing higher levels of stress had stronger causal beliefs about stress. No differences were found for the presence of high cholesterol, diabetes, and family history (Table 5).

## 4 Personal causal attribution

Overweight/obesity, stress and hypertension were most often reported to be the most important risk factor for respondents themselves (Supplementary file, Table 3). Men and women had similar ratings, except that stress was the most commonly chosen risk factor for women, while overweight/obesity was the most commonly chosen for men.

### 4.1 Illness perceptions and preferences for health check test results

The respondents were more likely to choose to participate in a health check than opt out. They preferred test results including both everyday words and an overall assessment, to receive a letter with their test results, to have lifestyle recommendations included, and to get 30 min consultation time. Respondents were negative towards cost and waiting two weeks or more for their test results. All illness representations and causal beliefs were tested for their influence on preferences for health check results. Three aspects of illness perceptions contributed to the model and were added in the final model: consequences, control, and personal causal attribution (family history). Respondents that perceived MI as having serious consequences on life placed higher value on receiving written information that included an overall assessment. Respondents that perceived they had low control over their MI risk, placed lower value on receiving lifestyle recommendations and were less negative to waiting three weeks for receiving their test results. Respondents that perceived family history as their most important cause for developing CVD were overall less positive to participating in a health check (Table 6).

**Table 6:** Respondents’ preferences for health check results and interactions with illness perception dimensions based on mixed multinomial logit.

Attribute levels		Coefficient	Standard error	95% CI	
Written results					
Lab results (ref)	Mean	−0.55	0.12	−0.34	−0.80
Lab results + everyday words	Mean	0.06	0.08	−0.22	0.11
	SD	0.08			
Lab results + everyday words + overall assessment	Mean	0.51**	0.09	0.33	0.69
	SD	0.37**			

Notification					
Electronic journal online (ref)	Mean	−0.24	0.04	−0.12	−0.26
Electronic journal online + letter	Mean	0.19**	0.04	0.12	0.27
	SD	0.39**			

Waiting time					
2 days (ref)	Mean	0.33	0.09	0.90	0.56
1 week	Mean	0.13	0.07	−0.00	0.27
	SD	0.14			
2 weeks	Mean	−0.60**	0.08	−0.76	−0.44
	SD	0.01			
3 weeks	Mean	−0.26*	0.10	−0.46	−0.06
	SD	0.60**			

Lifestyle recommendations					
Not included (ref)		−0.79			
Included	Mean	0.79**	0.06	0.67	0.91
	SD	0.53**			

Consultation time					
No consultation time (ref)		−1.01	0.06	−0.67	−0.91
15 min consultation time	Mean	0.46**	0.05	0.36	0.56
	SD	0.13			
30 min consultation time	Mean	0.68**	0.05	0.56	0.78
	SD	0.38**			
Cost (0–120 E)	Mean	−2.91**	0.16	−3.24	−2.59
Constant specific B	Mean	0.37**	0.06	0.25	0.48
Opt-out	Mean	−1.87**	0.18	−2.22	−1.51
	SD	2.98**			

*Interactions*					
Consequences * Everyday words		−0.05	0.10	−0.24	0.14
Consequences * Everyday words & overall assessment		0.28**	0.11	0.07	0.49
Low control * Lifestyle recommendations		−0.19*	0.09	−0.37	−0.014
Low control * 1 week waiting time		−0.11	0.11	−0.33	0.11
Low control * 2 week waiting time		−0.03	0.13	−0.28	0.22
Low control * 3 week waiting time		0.39*	0.15	0.09	0.69
My cause family history * opt-out		1.32**	0.49	0.36	2.27

Significance at *5% **1% AIC: 1.098.

## 5 Discussion

In this study of members of the public, women had stronger causal beliefs about stress, and more often choose stress as their most important personal cause for CVD, in line with previous studies in patient populations (Al-Smadi et al., 2016). It is known that education, sex, and health literacy can influence knowledge and beliefs about CVD risk factors (Damman et al., 2014). Low health literacy was associated with weaker causal beliefs regarding smoking, hypertension, unhealthy diet and lack of physical activity, while educational level was not associated with any of the B-IPQ components. Weaker causal beliefs for hypertension, physical activity, obesity/overweight and smoking, were also found amongst participants in this study whom the risk factor applied. Since low health literacy is associated with a higher CVD risk (Lindahl et al., 2020), those in greatest need for intervention may face challenges regarding utilization of health information needed for self-management. Strengthening causal beliefs of this group is a potential strategy to promote healthy lifestyle changes. Health promoting interventions aiming at improving people’s CVD knowledge and beliefs should therefore consider both CSM and health literacy. However, one should also consider weaker causal beliefs relating to personal risk factors could be due to a result of unconscious psychological defenses, rather than knowledge gaps. Such tendencies are more evident for lifestyle risk factors, which may include self-chosen behaviors that the person enjoys (Klein and Helweg-Larsen, 2002).

On the contrary, stronger causal beliefs were found for persons who experienced high stress. One explanation for this could be that stress is a self-assessed experience while hypertension in many cases is rather symptom free and clinically assessed. Stress in that sense is similar to self-perceived general health, which has a major influence on how individuals perceive their CVD risk (Grauman et al., 2021).

In a study from 2002, only 51% of respondents from the public in several European countries were aware that high cholesterol increases CVD risk (Erhardt and Hobbs, 2002), while respondents in this study had strong causal beliefs about cholesterol and only 3% answered, “don’t know.” On the contrary, 23% of the respondents reported unawareness regarding the causal impact of diabetes on MI. Informing the public about the increased risk of CVD that diabetes might cause is thus recommended.

Respondents with low perceived control over their CVD risk were less interested in lifestyle recommendations, which corresponds to previous findings where perceived low control in diabetes patients was associated with worse self-care behaviors such as diet (Nie et al., 2018). In our study sample, low perceived control was associated with smoking, high stress and a high self-perceived CVD risk. Respondents that perceived family history of MI to be their most important cause were less willing to participate in health checks. It is possible that this finding reflects a belief that family history is not as modifiable as other risk factors. Placing causal attribution on factors that are not modifiable makes health checks less attractive, since they focus on possibilities for intervention. Previous studies have found associations between family history and perceived MI risk (Grauman et al., 2021). It could be assumed that people that perceive their risk of MI as high would be more willing to participate in a health check. However, our results emphasize that the underlying causal beliefs behind the perceived risk matter (in terms of being modifiable or not) for the willingness to attend a health check. The influence of general beliefs about the causal impact of family history on preferences was also tested but did not contribute to improving the overall model. This may be an indication of the distinction between people’s causal beliefs in general and beliefs about themselves, which in this study, seems more relevant to the respondents’ decisions. Improving general knowledge about risk factors may therefore not have the expected effects on participation in preventive interventions, if the individual does not apply that knowledge to him/herself. Since control and causal attribution seems to impact on the willingness to receive lifestyle recommendations and attend health checks, it plays a crucial role in health check context, where the goal is to detect risk factors and promote healthy lifestyle. Risk communication based on CSM proved to alter diabetic patients CVD risk perception, however, the effect was only short term, indicating that communication must be repeated (Welschen et al., 2012). Another intervention successfully influenced MI patients' inaccurate and negative perceptions about MI, including increasing their perceived control and their awareness of multifactorial CVD risk factors, through individualized communication based on patients’ responses on the Illness Perception Questionnaire (Petrie et al., 2002). This approach may be adapted and applied as educational components included in health check interventions. When reaching the healthy public and altering their beliefs, the health information, should be based on common misperceptions and adapted to the groups with lowest health literacy and highest need of intervention, and on evidence based recommendations on risk communication (Fischhoff et al., 2011). An important message is that CVD risk is modifiable by personal efforts through lifestyle changes, and that health checks are effective at detecting CVD risk factors in people that otherwise are symptom free. Furthermore, CVD risk is multifactorial with modifiable risk factors, which may be even more important for a person who has a family history of CVD.

### 5.1 Strengths and limitations of the study

The study used cross-sectional data, which prohibits the ability to draw causal conclusions about the findings. The fact that the majority of the study population was highly educated and born in Sweden also limits the generalizability of the descriptive analysis. However, the lack of a representative sample does not necessarily influence the internal validity (associations between variables) (Rothman et al., 2013). The study consisted of a random sample of the public and adjusted for education, sex and age, which is a strength of the study. We asked about MI as an illness, in line with public conceptualisations while it is more accurately described as an acute symptom of a chronic underlying disease, which might have introduced confusion. However, the survey was pilot tested to avoid misinterpretations and previous studies^22^ have used the same wording, which informed the approach taken in the current study.

We also tested risk perception as an interaction with lifestyle recommendations, to explore its influence on preferences. High risk perception was associated with less willingness to receive lifestyle recommendation as personal control (result not shown). However, risk perception could not be included in the same model as control due to correlation issues. Future studies could investigate how risk perception might further influence the associations between B-IPQ, causal beliefs and preferences.

### 5.2 Conclusion

Illness perceptions differed due to presence of risk factors, age, sex and health literacy. Furthermore, illness perceptions influenced preferences for health check test results and willingness to participate in health checks. Illness perceptions should therefore be addressed when designing health communication and preventive interventions such as health checks, and methods for promoting accurate illness perceptions should be developed.

Funding

This study was funded by a grant from the Swedish Heart and Lung Association [grant number: 20150049].

## CRediT authorship contribution statement

**Åsa Grauman:** Conceptualization, Investigation, Data curation, Methodology, Formal analysis, Project administration, Software, Visualization, Writing – original draft. **Jennifer Viberg Johansson:** Conceptualization, Writing – review & editing. **Marie Falahee:** Conceptualization, Writing – review & editing. **Jorien Veldwijk:** Conceptualization, Methodology, Supervision, Writing – review & editing.

## Declaration of Competing Interest

The authors declare that they have no known competing financial interests or personal relationships that could have appeared to influence the work reported in this paper
