# Supplementary material for: Public perceptions of myocardial infarction: Do illness perceptions predict preferences for health check results
Source: Prev Med Rep. 2022 Jan 24;26:101683. doi: 10.1016/j.pmedr.2021.101683 (PMC8802064; doi:10.1016/j.pmedr.2021.101683)
Supplement: Supplementary Data 1 [file mmc1.docx]

## Supplementary file for article: Public perceptions of myocardial infarction: Do illness perceptions predict preferences for health check results, by Grauman Å, Viberg -Johansson J, Falahee M, Veldwijk J.

| **Which one of the following checkups do you prefer, Health checkup A or Health checkup B?**  (1 of 15) | |
| --- | --- |
| **Health checkup A** | **Health checkup B** |
| **Written test results:**   - Numerical lab values - Every day language - Overall assessment | **Written test results:**   - Numerical lab values |
| **How you will be notified:**   - Through your electronic health record | **How you will be notified:**   - Through your electronic health record - and a letter |
| **Waiting time for your test results:**  2 weeks | **Waiting time for your test results:**  2 days |
| **Lifestyle recommendations:**  Yes, it is included | **Lifestyle recommendations:**  No, it is not included |
| **Consultation with a medically trained person:**  30 minutes | **Consultation with a medically trained person:**  0 minutes |
| **This will cost you:**  600 SEK | **This will cost you:**  150 SEK |
|  |  |
| Would you participate in a health checkup similair to the one you chose, if you received an invitation in real life? | |
| **Yes**, I would participate in real life | **No**, I would not participate in real life |

Figure 1. Example of choice task

**Table 1.** Illness representations. 1-10. Differences in illness perceptions dimensions due to sociodemographic factors and CVD risk factors presented with mean difference and p-value for categorical variables, and the correlation coefficient for continuous variables (age, BMI). N=421

|  | **Consequence** | **Timeliness How long are you ill after an MI** | **Control over the risk of experiencing a MI** | **Treatment control** | **Concern, worry** | **Coherence (understand)** |
| --- | --- | --- | --- | --- | --- | --- |
| **Age** | -0.041  (0.43) | 0.055  (0.30) | -0.07  (0.17) | 0.050  (0.34) | 0.10  (0.041) | 0.107  (0.033) |
| **Sex**  Female vs male | -0.28  (0.12) | -0.41  (0.052) | -0.004  (0.98) | 0.34  (0.16) | 0.28  (0.26) | 0.56  (0.030) |
| **Education** university | -0.03  (0.13) | -0.20  (0.35) | 0.39  (0.11) | -0.18  (0.46) | -0.48  (0.056) | 0.33  (0.21) |
| **Health literacy**  High | -0.12 (0.53) | -0.31  (0.176) | -0.10  (0.69) | 0.22  (0.39) | -0.80  (0.003) | 0.57  (0.038) |
| **Medical or caring training** | -0.41  (0.06) | -0.43  (0.099) | 0.53  (0.071) | 0.56  (0.55) | -0.13  (0.67) | 2.66  (<0.000) |
| **Risk**  **Perception**  High vs same or lower | 0.02  (0.94) | 0.13  (0.65) | -0.63  (0.037) | 0.07  (0.81) | 1.78  (<0.000) | 0.52  (0.123) |
| **Hypertension** | -0.11  (0.58) | 0.43  (0.066) | 0.08  (0.76) | 0.58  (0.03) | 1.07  (<0.000) | 0.40  (0.157) |
| **Cholesterol** | -0.37  (0.14) | -0.41  (0.16) | -0.42  (0.90) | 0.47  (0.16) | 0.61  (0.083) | 0.65  (0.072) |
| **Diabetes** | -0.07  (0.84) | 0.10  (0.017) | 0.39  (0.40) | 0.17  (0.72) | 0.80  (0.10) | 0.26  (0.59) |
| **CVD** | -0.22  (0.52) | 0.13  (0.75) | -0.28  (0.55) | 1.2  (0.01) | 1.07  (0.026) | 1.24  (0.014) |
| **Fam history of MI** | -0.43  (0.036) | 0.37  (0.129) | -0.21  (0.45) | 0.09  (0.76) | 1.01  (<0.000) | 0.82  (0.006) |
| **Smoking** | -1.01  (0.02) | -0.29  (0.57) | -1.59  (0.004) | 0.43  (0.44) | 0.67  (0.24) | 0.37  (0.55) |
| **BMI** | -0.005  (0.93) | 0.018  (0.74) | -0.02  (0.66) | -0.032  (0.55) | 0.113  (0.023) | -0.040  (0.43) |
| **Stress**  high | 0.46  (0.32) | 0.62  (0.014) | -0.58  (0.037) | -0.05  (0.86) | 0.74  (0.013) | -0.32  (0.29) |

**Table 2.** Differences in causal attribution 1-5. Mean (SD), p-value below.

| **Cause** | **Mean (SD)** | **Do not know**  **n (%)** | **Age** | | **Sex** | | **Education** | | **Health literacy** | | **Presence of specific risk factor^a^** | |
| --- | --- | --- | --- | --- | --- | --- | --- | --- | --- | --- | --- | --- |
|  |  |  | **<57** | **>57** | **Female** | **Male** | **Low** | **High** | **Low/moderate** | **High** | **Yes** | **No** |
| **Smoking** | 4.4 (0.8) | 10 (2.4) | 4.27 (0.81) | 4.46 (0.73) | 4.41 (0.79) | 4.32 (0.76) | 4.36 (0.81) | 4.37 (0.73) | 4.31 (0.81) | 4.49 (0.68) | 3.75 (1.02) | 4.40 (0.75) |
| *P* |  |  | *0.016* |  | *0.223* |  | *0.926* |  | *0.025* |  | *>0.000* |  |
| **Hypertension** | 4.2 (0.8) | 10 (2.4) | 4.13 (0.79) | 4.17 (0.84) | 4.20 (0.84) | 4.11 (0.79) | 4.11 (0.92) | 4.21 (0.67) | 4.08 (0.86) | 4.31 (0.67) | 3.94 (0.85) | 4.23 (0.78) |
| *P* |  |  | *0.60* |  | *0.278* |  | *0.20* |  | *0.008* |  | *0.001* |  |
| **Overweight/obesity** | 4.2 (0.8) | 3 (0.7) | 4.17 (0.84) | 4.17 (0.76) | 4.13 (0.78) | 4.22 (0.80) | 4.20 (0.82) | 4.20 (0.77) | 4.12 (0.83) | 4.28 (0.72) | 3.91 (0.90) | 4.24 (0.76) |
| *P* |  |  | *0.97* |  | *0.272* |  | *0.724* |  | *0.065* |  | *0.002* |  |
| **High cholesterol** | 4.1 (0.8) | 13 (3.1) | 4.12 (0.85) | 4.13 (0.82) | 4.19 (0.81) | 4.06 (0.85) | 4.09 (0.85) | 4.16 (0.81) | 4.11 (0.85) | 4.17 (0.80) | 4.06 (0.77) | 4.14 (0.84) |
| *P* |  |  | *0.871* |  | *0.13* |  | *0.413* |  | *0.525* |  | *0.518* |  |
| **Stress** | 4.1 (0.8) | 12 (2.9) | 4.02 (0.89) | 4.15 (0.78) | 4.19 (0.77) | 3.99 (0.88) | 4.09 (0.82) | 4.08 (0.85) | 4.10 (0.84) | 4.08 (0.81) | 4.22 (0.81) | 4.04 (0.84) |
| *P* |  |  | *0.118* |  | *0.011* |  | *0.916* |  | *0.849* |  | *0.064* |  |
| **Unhealthy diet** | 4.0 (0.8) | 4 (1.0) | 4.05 (0.84) | 3.98 (0.82) | 4.08 (0.82) | 3.95 (0.83) | 3.96 (0.93) | 4.09 (0.68) | 3.951 (0.86) | 4.153 (0.73) | 3.80 (1.01) | 4.06 (0.78) |
| *P* |  |  | *0.341* |  | *0.123* |  | *0.102* |  | *0.021* |  | *0.018* |  |
| **Lack of physical activity** | 3.9 (0.9) | 5 (1.2) | 3.89 (0.87) | 3.89 (0.85) | 3.99 (0.87) | 3.79 (0.84) | 3.87 (0.86) | 3.91 (0.85) | 3.82 (0.87) | 4.030 (0.82) | 3.73 (0.91) | 3.97 (0.82) |
| *P* |  |  | *0.994* |  | *0.018* |  | *0.629* |  | *0.021* |  | *0.008* |  |
| **Heritage, it runs in the family** | 3.8 (0.9) | 12 (2.9) | 3.78 (0.93) | 3.85 (0.91) | 3.87 (0.91) | 3.75 (0.93) | 3.81 (0.93) | 3.81 (0.91) | 3.79 (0.97) | 3.86 (0.80) | 3.80 (0.92) | 3.82 (0.92) |
| *P* |  |  | *0.451* |  | *0.192* |  | *0.981* |  | *0.504* |  | *0.868* |  |
| **Diabetes** | 3.6 (0.97) | 98 (23.3) | 3.56 (1.01) | 3.64 (0.94) | 3.69 (0.92) | 3.51 (1.02) | 3.60 (0.99) | 3.59 (0.95) | 3.63 (0.95) | 3.53 (1.01) | 3.65 (0.98) | 3.60 (0.98) |
| *P* |  |  | *0.458* |  | *0.093* |  | *0.916* |  | *0.362* |  | *0.772* |  |
| **High alcohol intake** | 3.6 (0.9) | 38 (9.0) | 3.56 (0.95) | 3.61 (0.94) | 3.65 (0.93) | 3.53 (0.95) | 3.63 (1.00) | 3.53 (0.88) | 3.56 (0.95) | 3.66 (0.92) | - | - |
| *P* |  |  | *0.606* |  | *0.201* |  | *0.342* |  | *0.306* |  | - | - |
| **Ageing** | 3.3 (0.9) | 24 (5.7) | 3.23 (0.89) | 3.28 (0.93) | 3.20 (0.90) | 3.30 (0.92) | 3.18 (0.95) | 3.34 (0.87) | 3.23 (0.89) | 3.31 (0.96) | 3.23 (0.89) | 3.28 (0.93) |
| *P* |  |  | *0.610* |  | *0.271* |  | *0.083* |  | *0.391* |  | *0.610* |  |
| **Worry, sadness, loneliness** | 3.1 (1.1) | 43 (10.2) | 2.95 (1.11) | 3.29 (1.03) | 3.10 (1.09) | 3.15 (1.09) | 3.16 (1.11) | 3.08 (1.06) | 3.17 (1.10) | 3.03 (1.05) | - | - |
| *P* |  |  | *0.002* |  | *0.649* |  | *0.490* |  | *0.254* |  | - | - |
| **Virus/bacteria** | 2.4 (1.2) | 129 (30.6) | 2.26 (1.16) | 2.53 (1.15) | 2.41 (1.15) | 2.40 (1.18) | 2.51 (1.16) | 2.27 (1.14) | 2.45 (1.20) | 2.30 (1.08) | - | - |
| *P* |  |  | *0.045* |  | *0.934* |  | *0.073* |  | *0.305* |  |  |  |
| **Bad luck/chance** | 2.2 (1.0) | 60 (14.3) | 2.14 (1.00) | 2.29 (1.08) | 2.22 (1.09) | 2.21 (0.10) | 2.22 (1.02) | 2.19 (1.06) | 2.25 (1.06) | 2.14 (0.10) | - | - |
| *P* |  |  | *0.163* |  | *0.895* |  | *0.753* |  | *0.357* |  |  |  |

^a^Presence of risk factor for overweight/obesity and unhealthy diet were defined as having obesity. Presence of risk factor for lack of physical activity was defined as “never exercise.”

**Table 3**. Personal causal attribution; “The most important causes for my own risk of experiencing a MI”

| **The most important causes for my own risk of experiencing an MI** | **n** | **%** | **% within group with risk factor that placed it as nr. 1.** | **% within group with risk factor that placed it as top-three.** |
| --- | --- | --- | --- | --- |
| Overweight or obesity | 74 | 17.6 | 46% | 73% |
| Stress | 61 | 14.5 | 30% | 66% |
| Hypertension | 55 | 131 | 31% | 58% |
| Lack of physical activity | 41 | 9.7 | 20% | 42% |
| Family history | 37 | 8.8 | 23% | 57% |
| High cholesterol | 30 | 7.1 | 16% | 52% |
| Ageing | 30 | 7.1 | - | - |
| Bad luck | 22 | 5.2 | - | - |
| Unhealthy diet | 16 | 3.8 | - | - |
| Smoking | 10 | 2.4 | 38% | 76% |
| High alcohol consumption | 7 | 1.7 | - | - |
| Diabetes | 5 | 1.2 | 13% | 33% |
| Feeling down, feeling lonely, worry, emptiness | 5 | 1.2 | - | - |
| Virus or bacteria | 4 | 1 | - | - |
| Don’t know | 10 | 2.4 | - | *-* |
| Other | 14 | 3.3 | - | *-* |
